# 

**Published:** 1858-09

**Authors:** 


### OBITUARY.

Dr. L. G. Robinson, of Detroit, one of the editors of the *Independent*, an able writer, an accomplished scholar and able practitioner in the profession, died May 7th, at Detroit.

The world-renowned Prof. Robert Hare, of Philadelphia, died May 16th, 1858, in the seventy-fifth year of his age.

### RECEIPTS FOR JOURNAL UP TO OCTOBER 13th, 1858.

### Illinois.

| | | |
|---|---|---|
| Dr. A. C. Buffum, | vol. 1, 1858, | $2 |
| “ Sams & Hamilton, | “ “ | 2 |
| “ A. Taylor, | “ “ | 2 |
| “ A. F. Hand, | “ “ | 2 |
| “ J. W. McKinney, | “ “ | 2 |
| “ J. F. Marrh, | “ “ | 2 |
| “ T. D. Washburn, | “ “ | 2 |
| “ A. Hard, | “ “ | 2 |
| “ J. N. Allen, | “ “ | 2 |
| “ S. M. Reynolds, | “ “ | 2 |
| “ H. Noble, | “ “ | 2 |
| “ E. P. Cook, | “ “ | 1 |
| “ A. H. Smith, | “ “ | 2 |
| “ D. T. Kyner, | vol. 1 & 2, 1858-9, | 4 |
| “ J. F Daggett, | “ 6 & 1, 1857-8, | 4 |
| “ Wm. Hanly, | “ “ “ | 4 |
| “ K. E. Rich, | “ “ “ | 4 |
| “ Geo. Ryan, | “ “ “ | 4 |
| “ J. Keenan, | “ “ “ | 4 |
| “ C. Wheeler, | vol. 5, 6 & 1, 1856-7-8, | 6 |
| “ F. Blades, | “ “ “ | 6 |
| “ A. Eldridge, | vol. 4, 5 & half of 6, 1855-6 & — | 5 |

### Missouri.

| | | |
|---|---|---|
| Dr. Wm. G. Elder, | vol. 1, 1858, | $1 |

### Kansas.

| | | |
|---|---|---|
| Dr. C. A. Logan, | vol. 1, 1858, | $2 |

### Indiana.

| | | |
|---|---|---|
| Dr. W. T. Cornett, | vol. 1, 1858, | $2 |
| “ T. A. Graham, | “ “ | 2 |
| “ J. W. Hall, | “ “ | 2 |
| “ R. B. Denny, | “ “ | 2 |
| “ B. Kyler, | “ “ | 2 |
| “ R. D. Hutchinson, | “ “ | 2 |
| “ Samuel Daggy, | “ “ | 2 |
| “ W. Vannuys, | “ “ | 2 |
| “ Samuel Ross, | vol. 6, 1857, | 2 |
| “ T. P. Sellers, | vol. 6 & 1, 1857-8, | 4 |
| “ J. A. Berry, | vol. 6, 1 & half of 2, 1857-8 & — | 5 |

### Wisconsin.

| | | |
|---|---|---|
| Dr. H. D. Adams, | vol. 1, 1858, | $2 |
| “ S. C. Johnson, | “ “ | 2 |
| “ J. B. Dousman, | “ “ | 2 |
| “ James Pratt, | vol. 6 & 1, 1857-8, | 3 |
| “ D. La Count, | “ “ “ | 4 |
| “ Solomon Blood, | “ “ “ | 4 |

### Michigan.

| | | |
|---|---|---|
| Dr. Amos Crosby, | vol. 1, 1858, | $2 |
| “ Edwin Stewart, | “ “ | 2 |

### Iowa.

| | | |
|---|---|---|
| Dr. J. Doron, | vol. 1, 1858, | $2 |
| “ J. Williamson, | “ “ | 2 |

